# ISED: Constructing a high-resolution elevation road dataset from massive, low-quality in-situ observations derived from geosocial fitness tracking data

**DOI:** 10.1371/journal.pone.0186474

**Published:** 2017-10-13

**Authors:** Grant McKenzie, Krzysztof Janowicz

**Affiliations:** 1 Department of Geographical Sciences, University of Maryland, College Park, United States of America; 2 STKO Lab, Department of Geography, University of California Santa Barbara, Santa Barbara, United States of America; Beihang University, CHINA

## Abstract

Gaining access to inexpensive, high-resolution, up-to-date, three-dimensional road network data is a top priority beyond research, as such data would fuel applications in industry, governments, and the broader public alike. Road network data are openly available via user-generated content such as OpenStreetMap (OSM) but lack the resolution required for many tasks, e.g., emergency management. More importantly, however, few publicly available data offer information on elevation and slope. For most parts of the world, up-to-date digital elevation products with a resolution of less than 10 meters are a distant dream and, if available, those datasets have to be matched to the road network through an error-prone process. In this paper we present a radically different approach by deriving road network elevation data from massive amounts of in-situ observations extracted from user-contributed data from an online social fitness tracking application. While each individual observation may be of low-quality in terms of resolution and accuracy, taken together they form an accurate, high-resolution, up-to-date, three-dimensional road network that excels where other technologies such as LiDAR fail, e.g., in case of overpasses, overhangs, and so forth. In fact, the 1m spatial resolution dataset created in this research based on 350 million individual 3D location fixes has an RMSE of approximately 3.11m compared to a LiDAR-based ground-truth and can be used to enhance existing road network datasets where individual elevation fixes differ by up to 60m. In contrast, using interpolated data from the National Elevation Dataset (NED) results in 4.75m RMSE compared to the base line. We utilize Linked Data technologies to integrate the proposed high-resolution dataset with OpenStreetMap road geometries without requiring any changes to the OSM data model.

## 1 Introduction and motivation

In September of 2014, NASA’s Jet Propulsion Laboratory announced that it would be publicly releasing 1 arc second, or approximately 30m, resolution global (between 60° N and 56° S latitude) topographic data from NASA’s Shuttle Radar Topology Mission (SRTM) [1]. This release was heralded as a major success, significantly improving upon the previous global SRTM resolution of 3 arc seconds. Today, 1/3 arc second resolution elevation data is available for most parts of the U.S. via the United States Geological Survey’s National Elevation Dataset (NED) and selected regions are even available at 1/9 arc second. Through the advent of aerial Light Detection and Ranging (LiDAR) technology, the availability of high-resolution elevation data for specific regions has increased dramatically. Most of Oregon state, for example, has access to 9 foot resolution elevation data [2]. Slowly but steadily, the topography of the earth is being mapped at higher and higher resolutions.

Access to such high-resolution elevation data comes with a cost though. LiDAR data collection is expensive, time-consuming, and covers only a small region at a time. The complexity and cost often mean that the temporal resolution of the data is limited. Not surprisingly, most parts of the Earth surface can only be studied using 30m SRTM data. This resolution is sufficient for many large-scale applications, but falls short for small-scale purposes and more specifically for urban areas. Furthermore, the temporal resolution of the SRTM data is severely limited with the current dataset having been collected in 2000. Many domains and application areas would benefit from an alternative and inexpensive approach to constructing elevation datasets with high spatial and temporal resolutions.

To give a concrete example, in most counties in the United States emergency response personnel (EMP) are legally required to be able to access buildings within their service area. This implies that emergency response vehicles must be able to reach these buildings via the local road network. However, emergency response vehicles, e.g., fire trucks, are limited in their turning radius and ability to maneuver up an incline. In many U.S. counties it is explicitly mandated that roads in a region not exceed a certain grade, e.g., 10 percent [3] to allow access by EMP. Detailed data concerning the elevation and grade of many county roads is often unknown and even if high-resolution (LiDAR) data are available, buildings, transportation infrastructure, terrain features, and a dense vegetation canopy cover can often occlude the underlying roads. Furthermore, high-resolution data are not updated frequently even for some of the most developed areas.

Consequently, there is a need for alternative sources of elevation data. Intuitively one would assume that such an alternative source must be airborne, but this is not necessarily the case. Today, massive datasets are generated from cheap, sensor-rich devices operated by individuals that actively choose to share these data via online platforms. Social media platforms publish thousands of pieces of content per second from people that are opting to share not only opinions and photographs, but location information, personal physiological data, local environmental conditions, and so on. Wearable fitness trackers (e.g., Fitbit, Polar) have joined the social web and numerous applications have been developed to allow users of wearable devices to share their personal information with each other. Essentially, we are witnessing a shift from traditional *active* Volunteered Geographic Information (VGI), where users actively contribute spatial information, to *passive* VGI, where sensor-enabled devices passively share and communicate with other each other and through online services. In the following, we will argue that this enables entirely new means of collecting road network data including elevation, namely by in-situ sensing.

One application that is leading the *Online Social Fitness Tracking (OSFT)* revolution is *Strava* (http://www.strava.com). Strava is a fitness tracking application that allows users to upload completed activities to their platform and compete with other users over specific segments of roads or trails. In March of 2015 it was estimated that Strava had over 8 million users with roughly 1 million of those actively contributing data [4]. A unique feature of Strava is that users can upload activities (e.g., a bicycle ride) from virtually any mobile or wearable device that collects sensor information. This means that a million users openly share activity trajectories containing latitude, longitude, timestamp, and elevation data along with information pertaining to the device, gender, and age group of the contributor. Put differently, Strava has constructed a platform that collects and publishes hyper-local environmental and physiological sensor data, crowd-sourced from fitness enthusiasts. This information is already having an impact on domains ranging from health and fitness [5] to transportation infrastructure [6] and urban planning [7]. The company also currently offers a service aimed at urban planners, allowing those with access the ability to ingest their cycling data.

From a research perspective, these OSFT data offer an unprecedented opportunity to access high volumes of user-contributed, three dimensional data along the surface of road networks. In some cases, tens of thousands of users will have contributed three-dimensional fixes generated by cycling computers (e.g., Garmin Edge 500) to a single segment of road. While these data vary in their accuracy and precision, the sheer amount of data permits the opportunity to construct high-resolution elevation profiles for many of the world’s roads. In this work, we explore the possibilities for these data and show that it is feasible to construct low cost, high spatial and temporal resolution elevation profiles from user-contributed social fitness tracking data despite the fact that each individual observation may be of low quality. Furthermore we show that these data can be used to augment existing open geodatasets such as OpenStreetMap (OSM) through the addition of elevation values along ways and nodes. While we will use Strava as datasource here, our arguments and proposed methods are more generic and can be used to generate elevation data from any in-situ observations by citizens using smartphones and other forms of wearable technology.

**The concrete research questions addressed in this work are as follows**:

1. The quality of in-situ observations relies on the devices used and their sensors. Are the differences in vertical accuracy of devices that rely on barometric altimeters and those that do not, reflected in the data contributed to online social fitness tracking applications? Previous work has shown there to be significant differences in accuracy depending on the sensor availability of cycling computers. In this work we show the degree to which these differences in accuracy have permeated into the social fitness tracking application Strava.
2. Given the accuracy of certain types of sensors, what elevation accuracy can be expected from user-contributed cycling data? Through the removal of systematically erroneous data from devices lacking barometric altimeters, we show that it is possible to generate elevation profiles for road segments, accurate to within meters of ground-truth LiDAR data. Furthermore, we demonstrate that these data can be used to supplement existing approaches to producing high-resolution elevation profiles.
3. Can elevation data contributed by users of an online social fitness tracking application, be used to augment existing open geographic data platforms, e.g., OpenStreetMap? We demonstrate the feasibility of doing so by assigning elevation data aggregated from OSFT users to nodes along OpenStreetMap roads.
4. Finally, the elevation data constructed from OSFT users is of a high spatial resolution with elevation values every one meter along road segments. The inclusion of these data in OpenStreetMap is not directly possible as one would have to change the underlying OSM data model. Can high-resolution, user-contributed elevation dataset be constructed and linked back to OpenStreetMap data using Linked Data technologies [8]? We construct and publish a Linked Data version of our user-contributed elevation data and link it to LinkedGeoData (LGD) through sameAs relationships with LGD node identifiers. Thereby we make elevation data available and query-able without requiring any modifications to OpenStreetMap.

The remainder of this work is organized as follows. Section 2 introduces the data that are used in this research along with a description of the Strava API and relevant tools. Section 3 presents the methods that were used in generating the user-contributed elevation profiles. A number of points outlining the value of the user-contributed elevation profiles are described in Section 4 and the approach to augmenting existing open geodata is stated in Section 5. Finally, an overview of relevant and related work is given in Section 6 and conclusions and future work are presented in Section 7.

## 2 Data

In this section, we introduce the data, study areas, and the most frequent sensor platforms used by Strava users.

### 2.1 Segments & LiDAR data

Data accessible through the *Strava* platform is organized into two basic types of geospatial data. *Activities* are trajectories of geospatial information contributed by a single user over one continuous period of time (e.g., a bicycle ride). *Segments* are user-contributed portions of road or trail where athletes can compete for time [9]. In most cases, a single user activity will traverse one or many segments.

For our study, we selected five *high traffic* bicycle segments within Santa Barbara, California and Washington, District of Columbia. These segments vary in multiple ways as outlined in Table 1. For instance, *Segment A* is relatively long in distance with numerous uphill and downhill segments, while *Segment B* is shorter and involves a slow and steady incline. *Segment C* is a short, but unique segment that includes going under an overpass. Note that this segment is not included when comparing cycling segments to the ground-truthed LiDAR data for accuracy. This segment will be discussed further in Section 4.

**Table 1:** Segments selected to show a broad range of road types. Activity and Athlete counts current as of Oct 2016.

ID	Strava ID	Length	Elv. Diff.	#Activities	#Athletes	Description
Santa Barbara, CA						
**A**	2727695	4.5km	22.3m	24970	5197	Rural, Hilly, Canopy tree cover
**B**	749094	2.4km	47.9m	24347	4084	Sub-urban, Steady rise from sea
**C**	7324522	161m	2.4m	33805	5678	Urban, Under highway overpass
Washington, DC						
**D**	650024	1.6km	30.2m	18324	1949	Inner city park, hilly, tree cover
**E**	8068210	4.0km	13.1m	80995	8668	River’s Edge, tree cover

**Fig 1:**
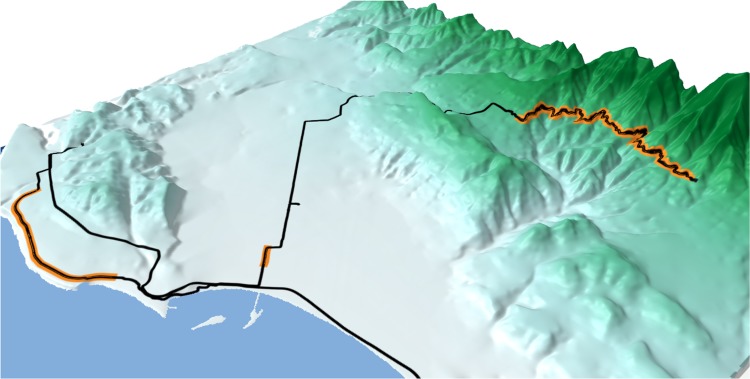
Santa Barbara study area showing a sample cycling activity (black) covering the three Strava segments (orange) and the LiDAR-based digital elevation model (green-scale gradient). Note that the LiDAR-based DEM has been smoothed for visualization purposes in this figure.

The selection of these three Santa Barbara segments was also based on the availability of an aerial LiDAR dataset used for ground-truthing the elevation values. Waveform LiDAR data were collected in August 2010 with a helicopter-mounted Riegl Q560 laser scanner. The data were georeferenced with two local differential GPS stations. The waveform was discretized and a bare earth digital terrain model was generated at 0.25m pixel resolution, later aggregated to 1.0m pixel resolution for the purpose of this study. The study area and relevant spatial layers are shown in Fig 1. In addition to the three Santa Barbara segments, two segments were chosen from within Washington, DC. These segments were included to ensure that any results of our analysis were not region-specific. Ground-truth elevation data for Washington DC was accessed from LiDAR data that was collected in the Winter of 2014 by Quantum Spatial Inc. using a Leica ALS 70 sensor. The data was accessed through a digital elevation model format at 1.0m pixel resolution. Combined, these five segments cover rural, sub-urban, and inner city areas and include various transportation and geographic features such as an overpass, hills, dense tree cover, and so forth.

### 2.2 Activities & devices

A sample of activities, each traversing at least one segment, were randomly selected from our segment set. A total of 11672, 11770, 15920, 15782 and 19231 activities were sampled from Segments A—E respectively with each activity consisting of a trajectory containing latitude and longitude point fixes along with elevation values (in meters to one decimal place) for each fix. In total, over 350 million *fixes* were accessed across all activities. Very little metadata is supplied on the contributing devices via the application programming interface (API), however the name of the device from which trajectory information was contributed was recorded. There were 70 unique devices used to collect activity data. Each device vendor’s specification website was accessed to determine which sensors are present. Table 2 lists the top 12 devices along with their activity count and sensor used for determining elevation. Just over 52% of the 74375 activities accessed where contributed via devices that contained barometric altimeter sensors, 40% relied on GPS elevation, and 8% could not be reliably determined.

**Table 2:** Top 12 devices used across three Santa Barbara segments.

Device	Activities	Elevation Sensor
Garmin Edge 500	29588	Barometric Altimeter
Strava iPhone App	29419	GPS
Strava Android App	8801	GPS
Garmin Edge 510	6415	Barometric Altimeter
Garmin Edge 800	4629	Barometric Altimeter
Garmin Edge 705	2732	Barometric Altimeter
Garmin Edge 810	2050	Barometric Altimeter
GPX File Upload	1574	Unknown
Garmin Edge 305	1541	Barometric Altimeter
Garmin Edge 520	1428	Barometric Altimeter
Garmin Forerunner 910XT	1346	Barometric Altimeter
Garmin Forerunner 305	1020	GPS

Through the Strava V3 API, the *efforts* for each of the segments were accessed. Each *effort* points to an *activity* in the sample set and includes the start and end indices and timestamps for when the activity traversed the specified segment. The fixes that traversed the segment were extracted (based on start and end indices) and stored as three dimensional point geometries in a PostGreSQL/PostGIS database. The median number of fixes per segment was calculated across all efforts in a given segment. Any segment effort containing a number of fixes less than two standard deviations from the median was removed from analysis. This was done to ensure that segment efforts with a very small number of fixes (e.g., 5) did not influence the aggregate value. The fixes from segment efforts that met this criteria were then joined as three dimensional line strings representing a single activity effort over a segment. Each of these activity line strings ranged in spatiotemporal resolution as the frequency with which coordinate fixes were recorded on the device was not uniform. However, every coordinate fix was accompanied by an elevation value regardless of the elevation sensor employed by the device.

## 3 Methods

While the previous section described the selection of the used data, we introduce the methods and processing steps employed to derive the final data product in the following.

### 3.1 2D segment data matching

**Fig 2:**
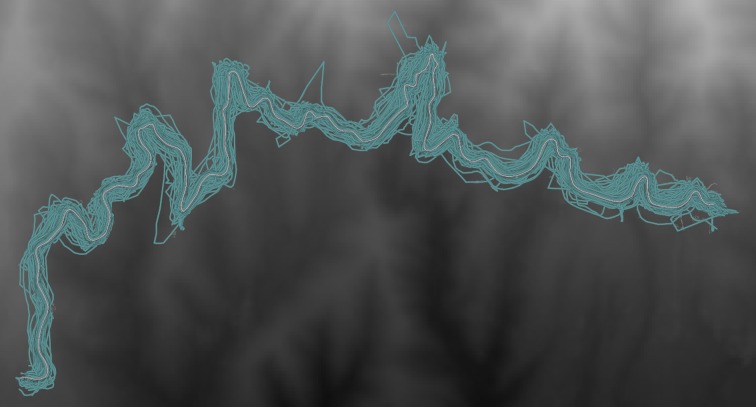
A sample of 10,000 activity efforts across Segment A (green). The OSM road segment is shown in pink. Base elevation data from USGS.

As a first step, we downloaded Polyline data from OpenStreetMap for the three Santa Barbara segments. These segments are subsections of the road network, which OpenStreetMap refers to as *ways*. Fig 2 shows a sample of these segment *efforts* in two dimensions. We calculated *Hausdorff distance* [10] between each individual activity effort and the OSM segment. Hausdorff distance calculates the similarity between two geometric objects. In this case, given the map projection, the unit of similarity is meters and roughly reflects the absolute maximum difference between segments. We assume that all athletes were cycling on the specified OSM road segment (and not off road) and that the OSM road segment represents the center line of the road. Table 3 reports the distance values for each of the Santa Barbara segments across all devices as well as the top three most common devices.

**Table 3:** Mean Hausdorff distance, in meters, for the three Santa Barbara segments. Standard deviation are shown in parentheses.

Segment	Garmin Edge 500	iPhone App	Android App	Overall
A	27.63 (8.77)	30.9 (13.94)	24.39 (10.25)	28.99 (11.49)
B	67.66 (25.62)	37.26 (22.12)	37.23 (14.73)	54.48 (29.43)
C	21.59 (19.36)	15.86 (8.75)	14.57 (10.64)	19.95 (18.05)

In examining these mean Hausdorff distance values we see that many of these segment efforts differ from the OSM center line by a significant amount. Much of this is likely due to obstruction and multipathing errors in the GPS units [11]. In some cases, especially in regions with tree canopy cover, there are significant jumps in consecutive location fixes. Existing work in this area has explored using these two dimensional activity efforts to better estimate road segments including combined work from Strava labs and OpenStreetMap [12, 13]. In this work, the Strava Labs team built a tool that iteratively loops through existing OpenStreetMap road segments and determines a function that best matches (snaps them to) existing *density-based center-lines* of the *Strava Heatmap*. The heatmap is a linear kernel density estimation based on all segment efforts from athletes that have contributed their activity data to the Strava application. Their result shows that high-quality data can be created from a massive dataset of low-quality fixes. Our work in this paper relies on this same fact and makes no effort to enhance their horizontal, two-dimensional approach, but instead focuses on the third dimension, namely *elevation*.

### 3.2 Elevation data

Multiple steps are required to generate 3D road profiles from in-situ observations. The first step involves ground-truthing and data cleaning. This is followed by a second step in which the elevation profiles are construed. After doing so, similarity is measured to compare the profiles based on three different measures, Root Means Square Error, Hausdorff Distance, and Earth Mover Distance. Based on this step, devices are selected that produce more accurate elevation readings. The results are compared to the LiDAR base line and the USGS National Elevation Dataset (NED).

#### 3.2.1 Ground-truthing & data cleaning

**Fig 3:**
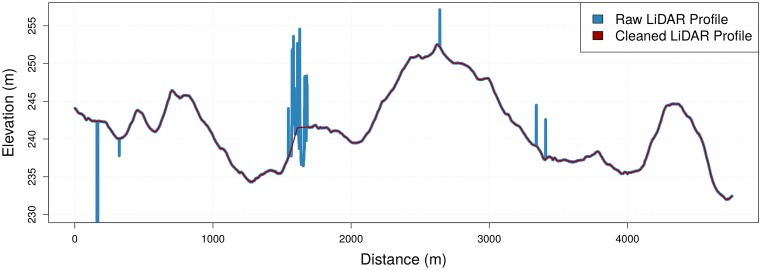
Cleaning LiDAR-derived profile errors to produce a smooth elevation profile.

As a first step in working with elevation data, each of the Santa Barbara OSM segment lines were converted to point representations by generating a single point every one meter along each segment. A one meter buffer was constructed around each point and used to clip the LiDAR digital elevation data. The minimum pixel elevation value was taken from each buffered region and assigned as the *true* elevation value for the point on the OSM road segment. The *minimum* pixel value within each buffer was taken to account for errors in elevation due to canopy cover at the exact point on the road segment. This approach worked in most cases, producing elevation profiles with reasonably smooth gradients across the entire road segment. In *Segment A* however, there were some issue with the LiDAR-derived elevation pixel values which produced a number of small errors in the elevation profile (Fig 3), likely due to canopy cover or angle of reflectance. Making the assumption that a paved road segment could not have a gradient greater than 30° over any one meter segment [14], these errors were rectified. First, we adjusting all elevation values that created an angle smaller or great than a 30° given the elevation value immediately before them to a value that makes an exact 30° gradient. A moving window containing five elevation values was averaged in these areas to further smooth the elevation profile. In Section 4 we show that the elevation profiles from the OSFT activities themselves can be used in lieu of this cleaning approach to provide a more accurate elevation profile.

#### 3.2.2 Constructing elevation profiles

Elevation profiles for each of the OSFT segment efforts were constructed based on the following method. Each segment effort is represented as a three dimensional line feature in PostGIS. Each line feature was generated by interpolating between, potentially sparse (greater than 1m), elevation fixes. Each node in an OSM road segment (now with nodes every 1m) is then used to extract the closest point on the line along each segment effort. The elevation at this point is then extracted from the three dimensional segment line feature. This method ensures that each segment effort returns an elevation value at the same one meter distance interval along the road segment. Finally, each segment effort is converted to a two-dimensional elevation profile with elevation (in meters) on the Y-axis and metric distance on the X-axis. Each of these elevation profiles is then compared to the respective OSM/LiDAR road segment elevation profile.

#### 3.2.3 Measuring similarity between effort data and LiDAR-derived OSM profiles

In order to compare elevation profiles, we must first introduce measures for assessing similarity. In this work, we employ three measures for comparing elevation profiles, namely *Root Mean Square Error (RMSE)*, *Hausdorff Distance (HD)* and *Earth Mover’s Distance (EMD)*. Each of these measures focuses on a different dimension of similarity and reporting the values together gives an overall holistic view of the similarity of two elevation profiles. The RMSE measures the square root of the average square of the difference between each elevation fix along a segment (every 1 meter). The HD measures how far apart two shapes are in metric space and in this case the measure reports the absolute maximum difference between the two profiles. Lastly, the EMD measures the similarity of two distributions, or in this case, two normalized elevation profiles by determining the *cost* of converting one profile into the other [15]. The smaller the value, the more similar the elevation profiles. Each of these measures was used to determine the similarity between each segment effort profile and the LiDAR-derived OSM elevation profile. In our case, the term *accuracy* is more appropriate than similarity as the OSM elevation values are based on ground-truthed LiDAR data and assumed to be the true elevation of the road segment.

#### 3.2.4 Segment effort attributes

**Fig 4:**
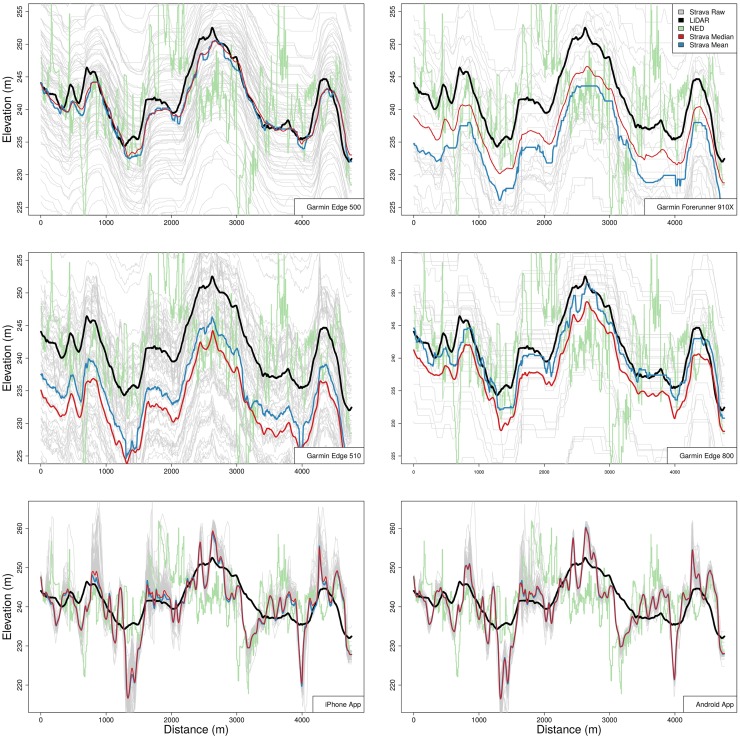
Elevation values for six devices over Segment *A*. The Garmin Edge and Forerunner devices all rely on barometric altimeter sensors to determine elevation while the iPhone and Android applications do not.

Before measuring the accuracy of these individual segment efforts, they are first split based on a variety of attributes. Elevation profiles from individual athletes are grouped (for example, one athlete traversed Segment *A* 493 times over 4 years) but no significant difference is found between individual athletes, gender, or age group. The profiles are also split by device with the most notable difference in accuracy found between devices that measure elevation through the use of a barometric altimeter sensor and those that do not. Fig 4 shows elevation profiles for six of the most common devices used in OSFT activities.

The Garmin Edge 500, 510, 800 and Garmin Forerunner 910X devices all use barometric altimeter sensors to determine elevation [16], while the iPhone and Android devices rely on alternative methods [17]. Though newer iPhone and Android devices do contain barometric sensors, the Strava application does not request access to this information. In general, devices relying on barometric altimeter readings tend to be precise and similar in shape to the LiDAR-based elevation data. The overall accuracy of each segment effort is quite low however, with elevation fixes differing by up to 60 meters at the same point along a road. Combining segment efforts within segments we find that the mean and median values are close to the OSM/LiDAR elevation profile for the Garmin Edge 500, but are less accurate for the other Garmin devices. There are a number of factors at work here, primarily the small sample size of 30 devices for the Forerunner 910Xs over *Segment A* versus the 4174 for the Edge 500. The RMSE between the LiDAR elevation and the median Garmin Edge 500 elevation is 3.37m with a HD of 6.43m and an EMD of 5.94*e*–05.

The non-barometric altimeter-enabled devices such as the iPhone and Android applications rely on a combination of location technologies, the primary method being global navigation satellite systems (GNSS) such as GPS-based elevation. The Strava support documentation, however, states that for “…devices without barometric altimeters, [strava] consults elevation databases to determine elevation at each point in the activity.” [18] The support documentation goes on to state that the database used in the United States is the *USGS National Elevation Dataset (NED)*, reporting elevation at 1/3 arc-seconds, the same dataset from which the green elevation profile is plotted in Fig 4. Notably, the mean and median elevation values, as well as the raw elevation values, shown in the plots for the iPhone and Android applications differ significantly from the interpolated NED profile shown in green. In fact the Hausdorff distance for the mean iPhone elevation to the NED profile is 24.8m. This implies that in this case either an alternate database is being used or that the elevation data from the device was not snapped to any database and instead GNSS elevation was reported unaltered. Either way, the non-barometric altimeter devices differ substantially from the LiDAR elevation data in Santa Barbara and should not be relied on for accurate elevation values. In comparison, the NED profiles for the Washington DC segments (D & E) are similar to the LiDAR reported elevation profiles on average, but still differ substantially in the Hausdorff distance measure. These differences are discussed further in Section 3.2.5.

#### 3.2.5 Refining the elevation model

Removing the non-barometric altimeter-enable devices from our set of segment efforts, we take the remaining efforts and calculate the overall mean and median elevation values for each of the three training segments (*A, B & D*). Remember that *Segment C* contains an overpass so is not used in the accuracy training models. *Segment D* was included as it represents a different region. To further refine the elevation model we split the segment efforts by device and year in order to determine if certain devices or software updates to devices produce higher accuracy mean and median elevation values. Any data prior to 2013 was excluded from our analysis as after splitting by device, there was not enough data to produce any meaningful results. In each of the training segments, we calculated the RMSE, HD and EMD between the OSM/LiDAR elevation profiles and the user-contributed segment effort median elevation profile across all barometric altimeter-enabled devices and years. The results of this analysis found that there was little difference between years (a proxy for device software updates) and that segment efforts from 2013-2015 should be included in the refined elevation model. In examining the accuracy of segment efforts from each of the barometric altimeter-enabled devices we found that Garmin Edge 500, 510 and 800 devices produced the most consistently accurate elevation profiles across the three training segments. This was likely due to these being the most popular devices used on the OSFT application and therefore had the least amount of variance across segments. Other devices such as the Garmin Forerunner 910XT produced very accurate results for *Segment B* but very inaccurate results for the other two training segments. Again, the amount of data produced by each of these devices likely had the strongest impact on accuracy. It should also be noted that number of activity efforts does have an impact on the median and therefore the overall accuracy. Across all training segments, we found that randomly reducing the number of activity efforts to below 110 began to have a significant negative impact on the overall accuracy and introduce high variance within each segment.

Combining segment efforts from the three top performing devices, namely the Garmin Edge 500, 510 and 800, we report the accuracy for all training segments as well as *Segment E*, a segment that was not used in the training data. Table 4 lists the three measures of accuracy for these four road segments. The elevation profiles calculated via the median of our user-contributed, in-situ elevation data (ISED) segment efforts are compared with those of the National Elevation Dataset provided by the U.S. Geological Survey [19]. Again, note that this NED is the source of elevation data that the Strava platform claims to use when a device does not have a barometric altimeter sensor. In all but one case, the ISED profiles are more accurate than the NED profiles and often by a large margin. In the case of *Segment D*, the RMSE of the NED is relatively low over the entire segment indicating high accuracy overall, but the maximum offset (HD) is over double that of the user-contributed median elevation. Furthermore, the shape (EMD) of the NED profile differs substantially from the ground-truth data relative to the ISED median. The number of activity efforts that contribute to the mean also have an impact on the overall accuracy.

**Table 4:** Three measures of elevation profile accuracy for four road segments. The median value for our in-situ elevation data (ISED) from user-contributed observations of each segment are compared to the interpolated National Elevation Data profile for the same segment.

Segment	Statistic	RMSE	HD	EMD
**A**	ISED Median	3.38	6.92	0.23
	NED	8.05	25.92	0.66
**B**	ISED Median	1.15	5.05	0.10
	NED	3.53	11.55	6.29
**D**	ISED Median	3.23	6.04	0.05
	NED	1.76	12.75	0.35
**E**	ISED Median	5.01	7.81	0.11
	NED	5.52	7.92	0.47

## 4 Supplementing and cleaning existing elevation data

Up to this point the focus of this research has been on constructing accurate user-contributed elevation profiles through comparison to existing high-resolution LiDAR data. However, an important benefit of these user-contributed elevation profiles is that they can contribute elevation profiles to regions where LiDAR data is either not available, inaccurate, or not suitable for determining elevation of a road. An example of the former is when part of a road segment has fully or partially closed canopy cover from vegetation suggesting that laser pulses are unable to breach the canopy and return true ground elevation values. An example of the latter is found when trying to construct an elevation profile for a road segment that passes below an overpass, *Segment C* for example.

Fig 3 depicts a concrete example in the LiDAR profile of *Segment A*. At a distance of roughly 1600 meters we see errors in the Raw LiDAR elevation profile which can be attributed to dense canopy vegetation cover that results in shorter LiDAR pulse returns or scattering based on leaf angle. This was initially cleaned to provide an accuracy comparison for the in-situ, user-contributed elevation profile based on the method discussed in Section 3.2. Since the ISED profiles are constructed from an aggregate of thousands of cycling activities and rely on barometric sensors, they are less prone to such canopy errors.

**Fig 5:**
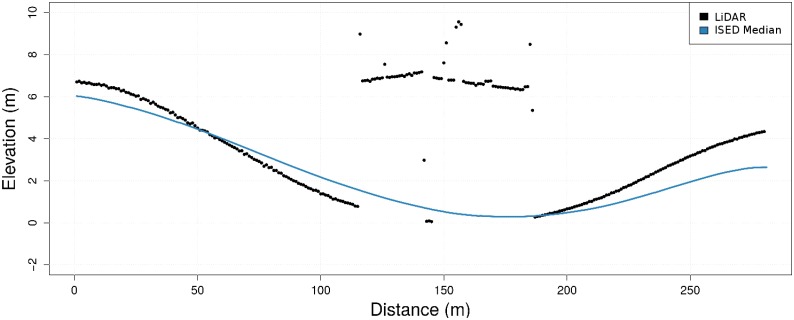
*Segment C* contrasting LiDAR elevation profile with ISED profile.

Additionally, ISED profiles can be used to supplement standard elevation profiling approaches in cases where elevation can not be determined from an areal view-point. *Segment C* is a road segment which passes under a highway overpass. As shown in Fig 5, the LiDAR data (black points) for this segment correctly reports a number of sudden elevation changes shown between 100 and 200 m along the X-axis, the overpassing highway. The blue line represents the median elevation reported from a reduced set of barometric altimeter enabled cycling devices along this segment. This line depicts a profile of the segment traversing under the highway overpass, unencumbered by the highway overpass, one that is not possible to recreate from aerial LiDAR scans.

The ISED approach can be employed in countless other situations where elevation cannot be accurately determined from airborne sources or the resolution of these source is inadequate. Furthermore, user-contributed elevation profiles can be generated across any time span, given a reasonable amount of data. For example, an elevation profile can be constructed for a road segment before and after a tectonic event to identify any major changes in slope or elevation. While this is possible with traditional elevation acquisition technology, repeated, high temporal resolution, data collection is often time consuming and cost prohibitive. ISED profiles offer an alternative and supplemental method to these traditional approaches.

## 5 Open elevation data

In this section we discuss how to make the created road network elevation data Web-available without having to change the OpenStreetMap data model.

### 5.1 Augmenting OpenStreetMap

Having demonstrated that high-resolution elevation data can be generated from in-situ observations from user-contributed OSFT data, we turn our focus to the process of publishing these data. OpenStreetMap (http://openstreetmap.org) is the largest and most comprehensive dataset of open geospatial data available today. The existing node and way structure of OSM uniquely identifies nodes along a street and the street segment, respectively (nodes and ways are also used to represent other point and line features). We use the identifiers associated with these nodes and ways as objects on which to link our user-contributed elevation data. The latitude and longitude coordinate geometry representations of these nodes are compared against our ISED 1m resolution road segments and the elevation value for each OSM node is determined by taking the elevation value from the closest ISED segment node. We recently updated OpenStreetMap (https://www.openstreetmap.org/changeset/46398492) to include elevation values for the sample segments described in this research and as we continue to generate user-contributed elevation values along new road segments, we will continue to update OpenStreetMap.

### 5.2 Linking high-resolution data

In most cases, OSM road segments are made up of far fewer nodes than the ISED 1m resolution road segments as their function is to trace the curvature and interconnectedness of roads, not to provide an even sampling of nodes. In the case of *Segment A*, for example, the overlap with OSM way #16249534 produces 3140 nodes in ISED *Segment A* and only 321 nodes for the equivalent OSM segment. Furthermore, these OSM nodes are typically not evenly distributed across the segment. Over all five segments in our sample dataset, we calculate an average RMSE of 6.83m, a HD of 43.42m and EMD of 0.036 between elevation profiles generated from the reduced set of OSM nodes and the higher-resolution ISED data. As reported by the EMD, the overall shape of the profiles remains similar, which is not surprising given that one is merely a reduced set of values from the other. The HD and RMSE values, however, point to some issues with reducing the dataset’s spatial resolution. While the RMSE between profiles is already approx. 7 meters, there are a number of instances where the differences are even more substantial.

**Fig 6:**
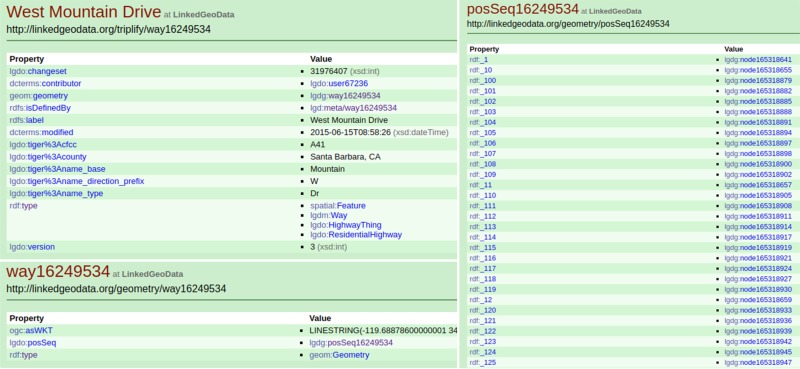
LinkedGeoData representation for part of *Segment A*.

Provided these findings, access to the higher-resolution dataset may be of interest to many domains and application areas. Rather than changing the OpenStreetMap data model by updating *ways* with nodes every 1m (which would essentially break the OSM data model for many other purposes and dramatically increase the data size), we decided to generate a supplementary dataset from ISED containing the higher horizontal resolution elevation data. Using Linked Data principles, we overlay an OSM *way* on our ISED road segment and assign it a new URI. This URI is then assigned a sameAs relationship to the OSM way in LinkedGeoData. LinkedGeoData.org [20] offers a structured, Linked Data [8] version of OSM data where each node and way are assigned a unique identifier (e.g., http://linkedgeodata.org/triplify/way16249534). A way represents a street segment and contains a geometry attribute which in turn links to a positional sequence object which lists a sequence of node URIs (Fig 6).

**Fig 7:**
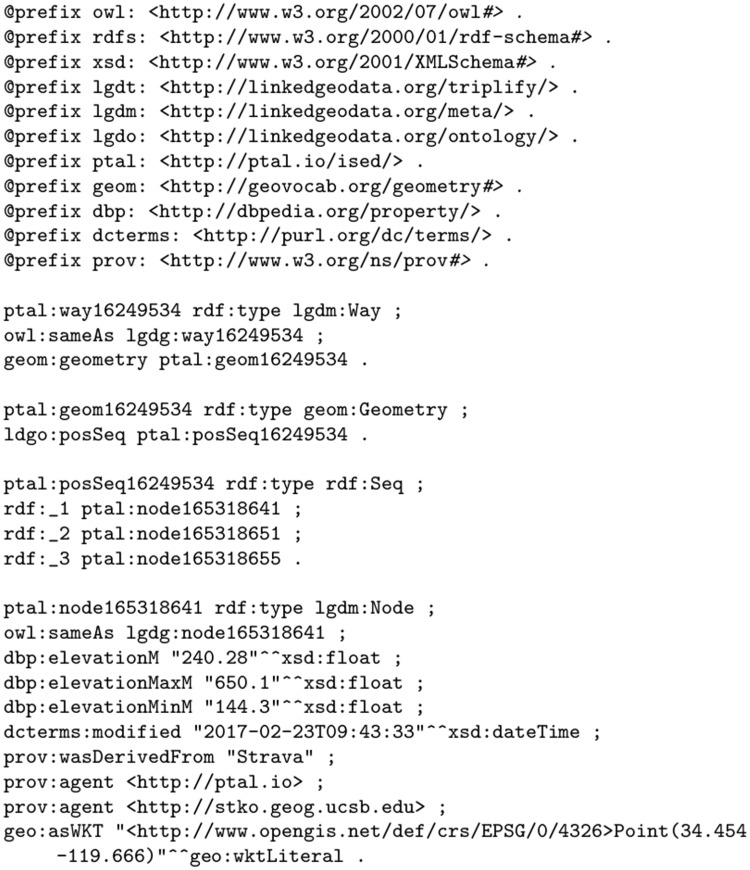
A sample of in-situ elevation data represented as linked data in turtle format. Note the sameAs relationships linking ISED entities (ways, nodes) to corresponding LinkedGeoData entities.

The *way* in our ISED consists of a sequence of nodes that, when appropriate, are linked back to the original OSM nodes. An example of these relationships are shown in Fig 7. A TTL file containing all segments used in this paper is accessible in RDF format at http://ptal.io/ised/santabarbara.ttl. Each node in our dataset consists of a required set of predicates as well as an optional sameAs predicate that links to a LinkedGeoData node. The required predicates are shown in Table 5.

**Table 5:** Predicates required of the ISED Node class.

Predicate	Prefix	Range
elevationM	http://dbpedia.org/property	float
elevationMaxM	http://dbpedia.org/property	float
elevationMinM	http://dbpedia.org/property	float
modified	http://purl.org/dc/terms	dateTime
wasDerivedFrom	http://www.w3.org/ns/prov	string
agent	http://www.w3.org/ns/prov	URI

## 6 Related work

Related work in this area has focused primarily on generating new datasets from user-contributed and crowd sourced data *or* on the accuracy and precision of local and global elevation datasets. To the best of our knowledge, little work has been done at the intersection of user-contributed elevation data from online social fitness tracking.

A number of recent projects have explored the sensors on mobile devices from a user-generated data perspective to generate a range of interesting datasets and services as well as research findings [21–24]. Our previous work [25] has shown that sensors accessible on most current smart-phones can be employed to differentiate place types and could be used in contribution to volunteered geographic services [26]. Microsoft’s *Nericell* project aimed at analyzing road and traffic conditions based on data collected via accelerometer and microphone sensors on mobile devices [27] while [28] measured urban noise through mobile devices’ sensors. Specific to the barometric sensor, existing work has identified this sensor in determining altitude estimations for indoor navigation [29, 30], medical applications [31, 32], and human movement and transportation research [33, 34].

As data sources and platforms, social fitness tracking and activity applications such as *Strava* have been the focus of quite a few previous publications [35]. Griffen et al. [36] relied on analysis of data from the Strava application to show the relationship between bicycling fitness and steep terrain while others explored the digital footprint of citizens based on their activity trajectories [37]. Online social fitness tracking applications have gone on to sell a lot of the fitness activity data contributed by their users for various purposes such as urban design [7, 38] and transportation infrastructure planning [6]. Systems have been designed for the purpose visualizing, sharing and analyzing much of this transportation data [39].

From an open geodata perspective, existing work has merged openstreetmap data with *Shuttle Radar Topography Mission (SRTM)* based digital elevation models (approx. 3 arc second resolution) to construct hydrological models [40]. The OpenStreetMap community itself has used SRTM data to construct the OpenCycleMap [41] an interactive web map which includes contour lines for bicycle routing. [42] has ambitiously merged various publicly available elevation datasets using SRTM as a gap-filler and published global elevation datasets at varying degrees of resolution. Recent work by Wang et al. [43] extracted elevation values from Google Earth and measured the accuracy for transportation applications. While their approach performs will when compared to benchmarks, there is no mention of the influence of canopy cover or other such obstacles. Furthermore, the frequency with which data is contributed to Strava means that our road elevation data can be updated daily (or in some cases hourly) and does not require an update from a third-party data provider.

## 7 Conclusions and future work

Generating high-resolution elevation profiles is very often costly and time consuming. For a number of applications in many parts of the world, the spatial and temporal resolution of existing elevation data is not sufficient. The recent rise of online social fitness tracking applications has allowed individuals to publish local elevation data by way of barometric altimeters and GPS sensors in their mobile and wearable devices. Although each individual in-situ observation varies substantially in terms of spatial and temporal resolution and accuracy, the extensive amount of data from a variety of devices invites the construction of an aggregate, up-to-date elevation dataset for road networks at a 1m spatial resolution. In this work, we have shown that elevation profiles generated from user-contributed data can approximate the accuracy of high resolution elevation profiles generated from ground-truthed LiDAR data. In fact, on average, our elevation profiles have a RMSE of 3.11m compared to the LiDAR data while using NED for the same profile results in a RMSE of 4.75m. Furthermore, we demonstrated that user-contributed elevation profiles can be used to supplement existing elevation data sources in situations where they fall short, e.g., in cases of overpasses. In contrast to LiDAR and NED data, our dataset can also be kept up-to-date by simply streaming in sensor readings from cyclists. Lastly, we introduce a method to enhance OpenStreetMap, an existing open geographic dataset, through the addition of elevation values along road segments. Using the tenets of Linked Data, we present an approach to publishing our high-resolution, user-contributed elevation data and linking them back to existing spatial data sources without having to change the OSM data model.

Future work in this area will involve expanding the scope of data sources from Strava cycling data to other platforms, e.g., *MapMyFitness* and other exercises, e.g., climbing. Efforts are currently underway to expand the regional scope of this work outside of the two study areas presented. One of the limitations of this work is that many of the regions that are in need of high resolution elevation models are places where uploading fitness tracking data is less common. We aim to explore the range of the various platforms and propose potential solutions for this limitation in future work. From a temporal perspective, next steps will focus on using in-situ elevation data to monitor changes in elevation and slope over time. Last, a RESTful application programming interface is in development that will return the elevation value of the closest known point provided geographic coordinates on the surface of the earth.
