# Correction: ISED: Constructing a high-resolution elevation road dataset from massive, low-quality in-situ observations derived from geosocial fitness tracking data

**DOI:** 10.1371/journal.pone.0225557

**Published:** 2019-11-14

**Authors:** Grant McKenzie, Krzysztof Janowicz

## Notice of republication

An incorrect version of Fig 2 was published in error. This article was republished on October 28, 2019 to correct for this error. Please download this article again to view the correct version.
